# Effect of provision of non-alcoholic beverages on alcohol consumption: a randomized controlled study

**DOI:** 10.1186/s12916-023-03085-1

**Published:** 2023-10-02

**Authors:** Hisashi Yoshimoto, Kyoko Kawaida, Shohei Dobashi, Go Saito, Yukiko Owaki

**Affiliations:** 1 Research and Development Center for Lifestyle Innovation, University of Tsukuba, 1-2 Kasuga, Tsukuba, Ibaraki 305-8550 Japan; 2 Department of Family Medicine, General Practice and Community Health, Institute of Medicine, University of Tsukuba, Tsukuba, Japan; 3 Department of Primary Care and Medical Education, Graduate School of Comprehensive Human Sciences, University of Tsukuba, Tsukuba, Japan

**Keywords:** Non-alcoholic beverages, Reduced alcohol consumption, Randomized controlled study

## Abstract

**Background:**

The use of alcohol-flavored beverages not containing alcohol (hereinafter referred to as non-alcoholic beverages) is recommended to reduce alcohol consumption. However, it is unclear if this reduces excessive drinking.

**Objective:**

To verify whether non-alcoholic beverages impact the alcohol consumption of excessive drinkers.

**Study design:**

Single-center, open-label, randomized, parallel-group study.

**Methods:**

Participants aged 20 years or older who were not diagnosed with alcoholism, who drank at least four times a week, and whose alcohol consumption on those days was at least 40 g in males and 20 g in females, were recruited. Participants were randomized into the intervention or control group by simple randomization using a random number table. In the intervention group, free non-alcoholic beverages were provided once every 4 weeks for 12 weeks (three times in total), and thereafter, the number of alcoholic and non-alcoholic beverages consumed were recorded for up to 20 weeks. The consumption of alcoholic and non-alcoholic beverages was calculated based on a drinking diary submitted with the previous 4 weeks of data. The primary endpoint was the change from baseline in total alcohol consumption during past 4 weeks at week 12. The participants were not blinded to group allocations.

**Results:**

Fifty-four participants (43.9%) were allocated to the intervention group and 69 (56.1%) to the control group. None of the participants in the intervention group dropped out, compared to two (1.6%) in the control group. The change in alcohol consumption was − 320.8 g (standard deviation [SD], 283.6) in the intervention group and − 76.9 g (SD, 272.6) in the control group at Week 12, indicating a significant difference (*p* < 0.001). Even at Week 20 (8 weeks after the completion of the intervention), the change was − 276.9 g (SD, 39.1) in the intervention group, which was significantly greater than − 126.1 g (SD, 41.3) in the control group (*p* < 0.001). The Spearman rank correlation coefficient between the change in non-alcoholic beverage consumption and alcohol consumption at Week 12 was significantly negative only in the intervention group (*ρ* =  − 0.500, *p* < 0.001). There were no reports of adverse events during the study.

**Conclusions:**

Providing non-alcoholic beverages significantly reduced alcohol consumption, an effect that persisted for 8 weeks after the intervention.

**Trial registration:**

UMIN UMIN000047949. Registered 4 June 2022.

**Supplementary Information:**

The online version contains supplementary material available at 10.1186/s12916-023-03085-1.

## Background

Excessive alcohol consumption is a global public health issue. In 2020, an estimated 1.34 billion people (1.03 billion males and 310 million females) consumed a harmful amount of alcohol [1], and more than 3 million people are dying from alcohol-related problems per year worldwide [2]. The World Health Organization (WHO) claims that excessive drinking not only causes health problems, including alcoholism, but also leads to other serious issues, including domestic violence and traffic accidents due to drunk driving. Thus, it is recommending evidence-based measures such as price increases and taxation on alcohol beverages, permitting only certified stores to sell alcohol, and strengthening penalties for underage drinking and drunk driving [3]. Japan is relatively permissive regarding drinking: for instance, there is an “all-you-can-drink” system in some restaurants [4], and alcoholic beverages are available for purchase 24 hours a day, every day throughout the year. The need for measures to improve this situation has been pointed out in an international report [5].

In Japan, efforts to reduce excessive drinking have been made through Health Japan 21, a program that constitutes Japan’s primary prevention strategy, and the enforcement of the Basic Law on Measures to Prevent Damage to Health Due to Alcohol, but their effects have been insufficient [5]. The “amount of alcohol that increases the risk of lifestyle diseases” is defined in Japan as pure alcohol consumption (hereinafter referred to as “alcohol consumption”) of 40 g/day or more in men and 20 g/day or more in women [6–8]. In 2019, it was reported that compared to 2010, the percentage of people who drank the above amounts did not increase or decrease in men and significantly increased in women [5]. One of the critical goals of Health Japan 21 is to reduce the number of people who drink alcohol in amounts that increase the risk of lifestyle diseases, and since this has not been achieved, it is necessary to take additional measures.

One strategy that has been discussed is the use of low-alcohol or non-alcoholic beverages [9]. Non-alcoholic beverages are alcohol-flavored beverages that contain no alcohol. They are alternatives to alcoholic beverages, and theoretically, if they replace alcoholic beverages, this may lead to public health benefits [10]. In its Global Strategy to Reduce Harmful Use of Alcohol, the WHO requests that the alcohol industry contributes to decreasing the harmful use of alcohol, for example by reducing the alcohol content of its products [11]. Rehm et al. identified the following three ways in which low-alcohol or non-alcoholic beverages may impact the harmful use of alcohol: (1) replacing standard alcoholic beverages with similar low-alcohol beverages may lead to the consumption of beverages with lower alcohol content without increasing the amount of liquid consumed; (2) replacing alcoholic beverages with non-alcoholic beverages for a certain period of time may allow drinkers to reduce their mean ethanol consumption; and (3) consumption of low-alcohol or non-alcoholic beverages may lead to the resumption of alcohol use by people who have been abstaining from alcohol [12]. Thus, non-alcoholic beverages can be an effective tool for reduction of alcohol consumption among excessive drinkers.

There is currently limited evidence on the effect of non-alcoholic beverages [13], with insufficient research on whether providing non-alcoholic beverages directly affects alcohol consumption. We examined this question in a randomized controlled study in excessive drinkers, excluding those with alcoholism. It is extremely valuable to verify effective methods for reducing alcohol consumption, not only for developing interventions for individuals, but also for formulating global strategies and community approaches such as policymaking.

## Methods

### Study design

This was a single-center, open-label, randomized, parallel group study.

### Participants

With reference to preceding studies [14, 15], the eligibility criteria for participants in this study were specified as follows: (1) at least 20 years of age and (2) drinking on 4 or more days per week, with alcohol consumption of at least 40 g for men or 20 g for women on each of the days. The exclusion criteria were consumption of non-alcoholic beverages at least twice per month, past history of liver disease, current pregnancy or nursing, alcoholism, lack of consent for the use of LINE® (a messaging application widely used in Japan that can be used on personal computers or smartphones; LINE Corp., Shinjuku-ku, Tokyo, Japan), and inability to understand the study explanation or answer the online survey conducted in advance, both of which were written only in Japanese. This study excluded individuals with alcoholism because it has been suggested that their use of non-alcoholic beverages may enhance alcohol craving and stimulate the desire to drink, which may increase the risk of drinking relapse [16].

### Setting

The survey was conducted from May 2022 to January 2023. Participants were recruited from May 30 to July 15, 2022, through the employee website of the University of Tsukuba, by putting up flyers inside and outside the University of Tsukuba campus, and by snowball sampling by the study personnel. The participants were randomly allocated to either the intervention or control group. People who wished to participate in the study were asked to answer an online questionnaire in advance to confirm eligibility and exclusion criteria, as well as to participate in an in-person 2-h briefing held on the University of Tsukuba campus and to provide written consent to participate. Nine briefings were held, and the number of attendees at each meeting was restricted to 15 as a COVID-19 prevention measure. In addition to interviews for alcoholism with physicians, measurement of height and weight and collection of saliva samples, this 2-h face-to-face orientation session prior to randomization included careful explanation about this study. We explained the effect of providing non-alcoholic beverages on alcohol consumption was unknown. We also asked participants in both groups to go about their daily lives as usual, with the exception of keeping a drinking diary.

### Procedures

The study procedures are described in Table 1. Week 0 of the study was defined as the start of the intervention. In the preparation period, the number of weeks was counted backwards, specifically Week (-4) and Week (-2). After the start of the intervention, the numbers of weeks were specified as Week 4, Week 8, and Week 12, and in the follow-up period, they were specified as Week 16 and Week 20. At Week (-4), a study briefing was held, including an interview with a physician experienced in the diagnosis and treatment of alcoholism to initially confirm eligibility criteria. Two physicians were in charge of interviews. Each individual was interviewed by one of these physicians, who independently formulated the diagnosis. All individuals who met the eligibility criteria underwent a group briefing, and consent to participate was obtained in writing. At this briefing, participants completed a questionnaire regarding basic attributes, information about drinking, and other factors; in addition, height and body weight were measured and a saliva test was administered to assess the activity of genes related to alcohol metabolism. After the briefing, the participants were randomized to either the intervention or control group by simple randomization using a random number table [17]. To prepare for the provision of the non-alcoholic beverages described below, at Week (-2), participants were told which group they were allocated to. During the post-allocation period (the intervention period, from Week 4 to Week 12) and the follow-up period, the participants in both groups were asked to record their consumption of alcoholic and non-alcoholic beverages in a drinking diary every day and to submit it every 4 weeks. After the briefing, the study participants were contacted only by phone or via internet. At the end of the study, a gift card worth 10,000 yen (approximately 73.67 US dollars) was given to all participants as a reward. In addition, each participant in the control group received up to five cases of non-alcoholic beverages of their choice.

**Table 1:**
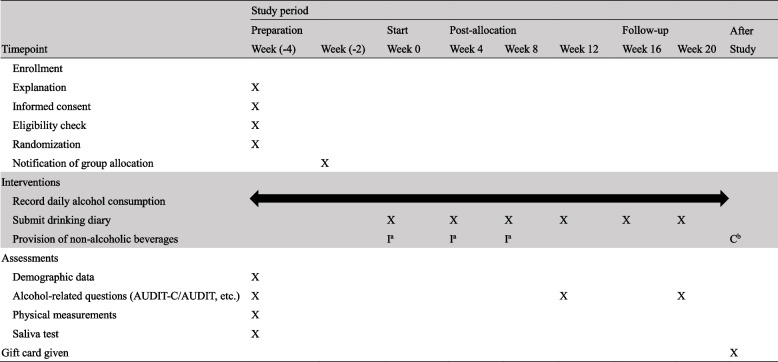
Study procedures

*I* intervention group, *C* control group, *X* all participants

^a^Up to 3 cases of non-alcoholic beverages were provided at a time; each contained 24 350-mL bottles according to each participant’s request

^b^Up to 5 cases of non-alcoholic beverages were provided, each containing 24 350-mL bottles according to each participant’s request

### Intervention

During the 12-week intervention period, free non-alcoholic beverages were provided once every 4 weeks (three times in total). Each case included 24 350-mL bottles. Up to three cases were provided at a time, with the exact number depending on each participant’s preference. Participants selected beverages from among 22 products from four manufacturers, specifically the six top-ranked beer-flavored products and the 16 top-ranked cocktail-flavored products according to the sales ranking in the Japanese market in 2021. The selected non-alcoholic beverages were shipped to participants’ homes by a package delivery company.

### Measurement

The self-administered questionnaire used in the briefing included questions regarding basic attributes such as age, sex, race, marital status, the highest level of education, employment status, household income, smoking history, and subjective view of health [18], as well as the Alcohol Quality of Life Scale (AQoLS) [19] and questions related to drinking, such as the Alcohol Use Disorders Identification Test (AUDIT) [20] and the number of binge-drinking episodes within the past 1 month. The definition of binge drinking varies across studies, but it commonly refers to the consumption of five or more drinks by men or four or more drinks by women over a period of about 2 h [21]. Since a standard drink is defined in Japan as 10 g of pure alcohol, we defined binge drinking as consumption of 50 g or more of alcohol by men and 40 g or more by women in a 2-h period on one occasion within the past month. Subjective view of health was one of the QOL indices. In response to the question “What do you consider to be your usual health condition?” the participants were asked to select one of four choices: “very healthy,” “fairly healthy,” “not so healthy,” and “not healthy.” The AQoLS is a scale for the evaluation of the effects of alcohol on health-related QOL. We used the Japanese version of the AQoLS, which, like the original, consists of 34 scientifically verified questions. AUDIT is a screening tool for alcohol use disorder developed by the WHO, and consists of 10 questions, with a full score of 40 points.

As genetic information related to alcohol metabolism, the gene activities of alcohol dehydrogenase 1B (*ADH1B*) and aldehyde dehydrogenase 2 (*ALDH2*) were examined using a saliva test. ADH1B is one of the enzymes that degrades ethanol, and ALDH2 is among those that degrades acetaldehyde. Differences in their activities are known to affect drinking behavior [22].

The drinking diary was designed in a calendar format, in which the amount of alcoholic and non-alcoholic beverages consumed were to be entered under each date. The drinking diary was provided to the study participants in printed form or in a Microsoft® Excel® file according to their choice, and they were asked to submit it to the study secretariat once every 4 weeks, either in an e-mail attachment or by LINE® (whereby photos of the filled-out diary were attached). For alcoholic beverages, participants were asked to specify the type, alcohol content, and the amount of consumption in order to calculate alcohol consumption, and the number of episodes of heavy episodic drinking (HED) was also counted. HED was defined as the consumption of at least 60 g of pure alcohol on at least one occasion in the past 30 days [2].

### Outcome

The primary endpoint was the change in total alcohol consumption from baseline during the past 4 weeks at Week 12 [23, 24]. Alcohol consumption (grams) was calculated based on the drinking diary data using the following formula: “consumption (mL) × alcohol concentration (%, v/v) × specific gravity (0.8)/100” [15]. In addition, the change from baseline in the consumption of non-alcoholic beverages was calculated based on the amount (mL) of non-alcoholic beverages consumed as recorded in the drinking diary. Furthermore, to examine whether alcohol consumption was reduced by replacing alcoholic beverages with non-alcoholic ones, the correlation between changes in alcohol consumption and the consumption of non-alcoholic beverages was analyzed.

### Sample size

The necessary sample size was calculated based on the effect size of the change in alcohol consumption (Cohen’s *d* = 0.59, unpublished data) determined in a preliminary investigation we performed by providing a brief intervention (a quick counseling by healthcare personnel) to excessive drinkers and comparing the outcomes with a control group, with a significance level of 0.05 and a statistical power of 0.80. Forty-seven participants per group and 94 in total (two groups) were determined to be necessary. Taking into consideration possible participant dropout during the intervention period, approximately 25% of 94, i.e., 26, was added, and 120 was specified as the target number of participants.

### Randomization and blinding

A random number table was used to perform simple randomization and to select the number of participants in the intervention group. After the eligibility and exclusion criteria were confirmed and consent was obtained, participants were allocated into either the intervention or control group and notified of the randomization results via e-mail within 2 weeks after the briefing. Allocation was performed by Investigator K, without disclosing the allocation or its order to the other investigators. Because people can choose their preferred non-alcoholic beverages in their everyday lives, and since the dropout rate might be increased if participants were provided with beverages that they did not like, those in the intervention group were not blinded so that they could choose their non-alcoholic beverages. For this reason, no items to facilitate blinding were provided to the control group participants during the study, but instead non-alcoholic beverages were provided after the study period had ended. The drinking diary data were evaluated by Investigators K, S, and O, who were blinded to the allocations of participants in both groups. Investigator D was not involved in briefings, randomization, questionnaire development, or the evaluation of drinking diary data, and therefore was able to perform data analysis in a blinded manner.

### Statistical methods

The available data of all participants were included according to the original allocation in an intention-to-treat analysis. The unsubmitted data of the participants who dropped out were treated as missing data. The normality of the data was evaluated by the Kolmogorov–Smirnov test. Intergroup comparisons of baseline data were performed by *t*-test in cases of normal distribution and by the Mann–Whitney *U* test in cases of non-normal distribution. The chi-square test or Fisher’s exact probability test was performed for categorical variables. Two-way analysis of variance (ANOVA) was used to analyze changes from baseline in the consumption of alcoholic and non-alcoholic beverages in the two groups, using group and time as factors. Normality of the data was not observed for either variable. However, homogeneity of the variance of the data was confirmed for both variables using Levene’s test, and therefore, the results of the analysis of variance were accepted with reference to a preceding study (*n* = 121) [25].

As post hoc tests, Dunnett’s test was performed to compare temporal sequences within the same group with reference to Week 4, and Bonferroni’s test was performed to analyze intergroup differences at individual time points. Correlations were evaluated based on Spearman’s rank correlation coefficients. The significance level was 5%. Stata 17.0 for Windows (Stata Corp., College Station, TX, USA) and GraphPad Prism v. 9.0 (GraphPad Inc., La Jolla, CA, USA) were used for all analyses.

## Results

**Fig. 1:**
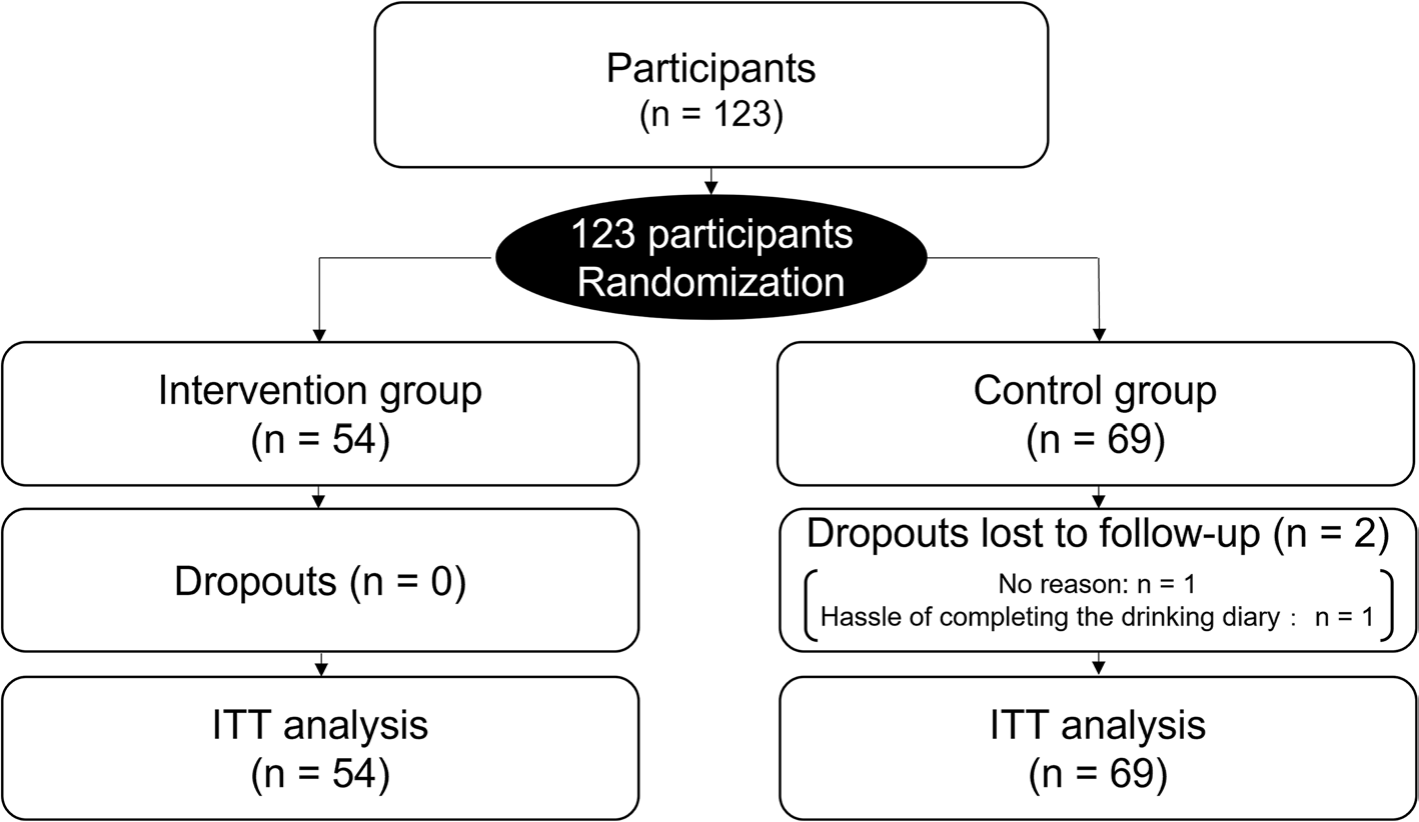
Study flow diagram

The study flow chart is shown in Fig. 1. One hundred twenty-three people wished to participate in the study and all of them were randomized. Fifty-four (43.9%) were allocated to the intervention group and 69 (56.1%) to the control group. None of the participants in the intervention group dropped out, compared to two participants (1.6%) in the control group. One participant did not specify the reason for dropping out, while the other cited the hassle of filling out the drinking diary.

Table 2 and Table S1 show the basic attributes of all 123 participants. There were no significant differences between the intervention and control groups in age (22 to 72 years), sex, race, height, body weight, marital status, highest level of education, employment status, household income, smoking history, AUDIT score, AQoLS score, subjective view of health, or number of binge-drinking episodes. Data on polymorphisms of the *ADH1B* and *ALDH2* genes, obtained from 122 participants, did not differ significantly between the two groups (Table S2).

**Table 2:** Characteristics in the participants

	*n* = 123	Intervention group *n* = 54 (43.9%)	Control group *n* = 69 (56.1%)	*p*
Age (years, SD)	47.5 (10.2)	47.8 (10.7)	47.2 (9.8)	0.74^a^
Female (number of participants, %)	69 (56.1)	28 (51.9)	41 (59.4)	0.40^b^
Highest level of education (number of participants, %)				
Junior high school	1 (0.8)	1 (1.9)	0 (0.0)	0.15^c^
High school	19 (15.5)	13 (24.1)	6 (8.7)	
Vocational school	12 (9.8)	4 (7.4)	8 (11.6)	
Junior college, specialized vocational high school	17 (13.8)	6 (11.1)	11 (15.9)	
College	50 (40.7)	22 (40.7)	28 (40.6)	
Graduate school	24 (19.5)	8 (14.8)	16 (23.2)	
AUDIT (points, median, IQR)	9.0 (7.0)	11.0 (7.0)	8.0 (7.0)	0.23^d^
Binge drinking (times/month, median, IQR)	4.0 (13.0)	4.0 (9.0)	3.0 (13.0)	0.53^d^

*SD* standard deviation, *IQR* interquartile range

^a^*t*-test, ^b^Chi-square test, ^c^Fisher’s exact probability test, ^d^Mann–Whitney *U* test

Alcohol consumption, the consumption of non-alcoholic beverages, and the number of instances of HED are shown in Table 3 as baseline data. The median baseline alcohol consumption was 996.0 g (interquartile range [IQR], 825.0) in the intervention group and 887.5 g (IQR, 765.0) in the control group, indicating no significant difference (*p* = 0.39).

**Table 3:** Baseline alcoholic and non-alcoholic beverages consumption and the number of heavy episodic drinking

	*N* = 122	Intervention group, median *n* = 54 (44.3%)	Control group, median *n* = 68 (55.7%)	*p*
Alcohol consumption (g/4 weeks, IQR)	935.5 (771.0)	996.0 (825.0)	887.5 (765.0)	0.39^d^
Non-alcoholic beverage consumption (mL/4 weeks, IQR)	0.0 (350.0)	0.0 (700.0)	0.0 (0.0)	0.064^d^
Number of HED (times/4 weeks, IQR)	6.0 (12.0)	6.0 (16.0)	6.0 (11.0)	0.92^d^

*HED* heavy episodic drinking, *IQR* interquartile range

^a^*t*-test, ^b^Chi-square test, ^c^Fisher’s exact probability test, ^d^Mann–Whitney *U* test

**Fig. 2:**
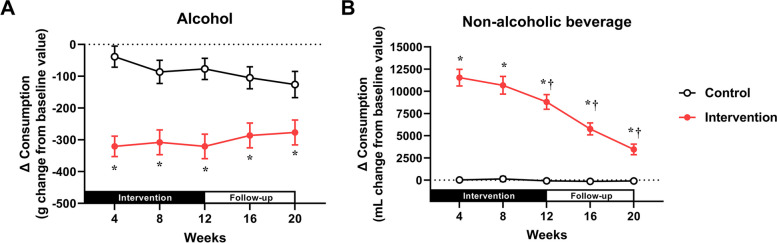
Changes in alcoholic and non-alcoholic beverage consumption from baseline. **A** Alcohol consumption (interaction of group and time: *p* = 0.040, *η*^2^ = 0.006; main effect of group: *p* < 0.001, *η*^2^ = 0.125; main effect of time: *p* = 0.846, *η*^2^ = 0.001). **B** Non-alcoholic beverage consumption (interaction of group and time: *p* < 0.001, *η*^2^ = 0.057; main effect of group: *p* < 0.001, *η*^2^ = 0.425; main effect of time: *p* < 0.001, *η*^2^ = 0.063). *p < 0.05 vs. the control group at the same time point. ^†^*p* < 0.05 vs. Week 4

The longitudinal changes in alcohol consumption and non-alcoholic beverage consumption relative to baseline at Weeks 4, 8, 12, 16, and 20 are presented in Fig. 2. Two-way ANOVA of the change in alcohol consumption using group and time as factors indicated a significant interaction (*p* = 0.040, *η*^2^ = 0.006, Fig. 2A). The result of Bonferroni’s test for multiple comparisons indicated significantly lower values in the intervention group than in the control group at all time points from Week 4 to Week 20 (*p* < 0.05 for all). The main outcome in this study, the change in alcohol consumption at Week 12 (mean ± standard deviation), was − 320.8 ± 283.6 g in the intervention group compared with − 76.9 ± 272.6 g in the control group, while that at Week 20 was − 276.9 ± 287.6 g in the intervention group compared with − 126.1 ± 338.4 g in the control group. Neither group showed a significant effect of time on the change in alcohol consumption. Two-way ANOVA of the change in consumption of non-alcoholic beverages also revealed a significant interaction (*p* < 0.001, *η*^2^ = 0.057, Fig. 2B). Bonferroni’s test for multiple comparisons indicated significantly higher values in the intervention group than in the control group at all time points from Week 4 to Week 20. Dunnett’s test for multiple comparisons indicated no significant difference between Week 4 and Week 8 in the intervention group, but significantly lower values than those at Week 4 were observed at each time point from Week 12 onward (*p* < 0.05 for all).

**Fig. 3:**
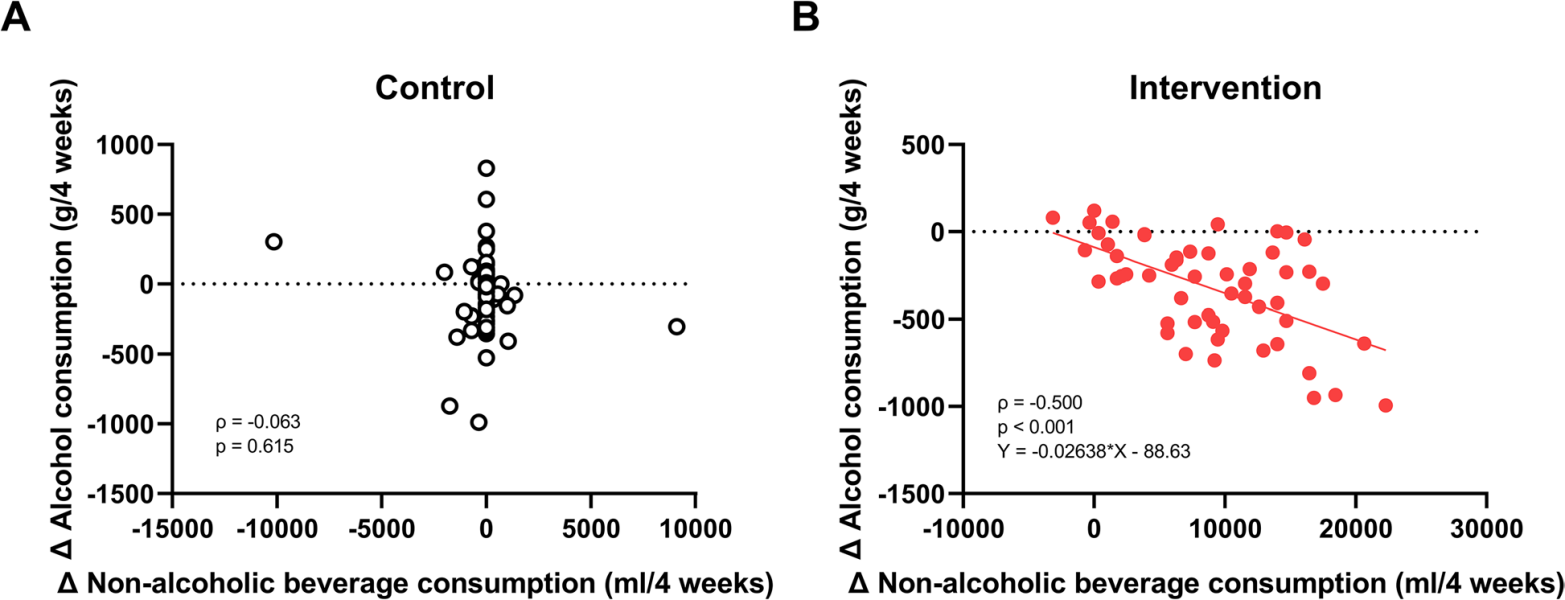
Correlation between changes in alcohol consumption and non-alcoholic beverage consumption at Week 12 in the control and intervention groups. **A** Control group (*n* = 67). **B** Intervention group (*n* = 54)

Spearman’s correlation coefficient was used to analyze each group in terms of the changes from baseline in both alcohol consumption and non-alcoholic beverage consumption at each time point from Week 4 to Week 20. The main outcome, alcohol consumption at Week 12, was not significantly correlated with non-alcoholic beverage consumption in the control group (*ρ* =  − 0.063, *n* = 67, *p* = 0.615). In contrast, a significant negative correlation was noted in the intervention group (*ρ* =  − 0.500, *n* = 54, *p* < 0.001, Fig. 3) and was also observed at Weeks 4, 8, and 16, with no significant correlation at Week 20 (Fig. S1).

No adverse events were reported during the study. The total cost of the non-alcoholic beverages provided to the intervention group, including shipment costs, was 1,061,564 yen (approximately 7820.6 US dollars), which equated to a mean of 111.70 yen (approximately 0.82 US dollars) per 350-mL bottle.

## Discussion

In this study, the provision of free non-alcoholic beverages for 12 weeks significantly reduced alcohol consumption. In the intervention group, this effect persisted not only during the intervention period but also up to 8 weeks following completion of the 12-week intervention. Our study is the first to suggest that providing non-alcoholic beverages reduces alcohol consumption.

At Week 12, alcohol consumption was reduced by an average of 11.5 g per day. On the other hand, the average volume of non-alcoholic beverages consumed per day was 314.3 mL. Correlation analysis showed a significant and moderate negative correlation between changes from baseline in alcohol consumption and non-alcoholic beverage consumption, but only in the intervention group. This suggests that the reduced alcohol consumption in the intervention group may have been caused by the replacement of alcoholic beverages with the provided non-alcoholic beverages. A previous study demonstrated that greater availability of non-alcoholic beverages increased the odds of selecting non-alcoholic beverages rather than alcoholic beverages [26]. In our study, this increased availability presumably resulted from the delivery of non-alcoholic beverages to participants’ homes. It has been pointed out that replacing alcoholic with non-alcoholic beverages may lead to public health benefits [10], and therefore, such replacement may be effective as one method for reducing the harmful impact of alcohol.

However, the correlation between the changes in alcohol consumption and non-alcoholic beverage consumption in the intervention group gradually weakened after Week 8, and the significant correlation between them disappeared at Week 20. Although the reason remains unclear, it is possible that some of the non-alcoholic beverages provided at Weeks 0, 4, and 8 were consumed by the participants in the intervention group even after Week 12. Thus, although the effect of replacing alcoholic beverages lasted for a certain period, it was weakened when all the available non-alcoholic beverages were consumed. While the participants were not restricted from purchasing either alcoholic or non-alcoholic beverages throughout the study period, we could not distinguish whether the consumed non-alcoholic beverages were provided or voluntarily purchased because our information from the participants pertained only to the consumption amount. Thus, further studies are warranted to clarify this hypothesis. Nevertheless, the reduction of alcohol consumption in the intervention group was not affected by time and was still observed at Week 20 (8 weeks after completion of the intervention). This suggests that a certain degree of behavioral modification may have occurred, in addition to the effect of replacing alcoholic with non-alcoholic beverages, and this may have contributed to the persistent reduction in alcohol consumption in the intervention group after the intervention was complete. As this study was not conducted in a blinded manner, the Hawthorne effect, which refers to behavioral changes driven by attention or expectation from others, including investigators, may have affected the results [27]. However, no Hawthorne effect occurred in a preceding study in which alcohol consumption data were collected by an online survey [28]. To prevent this effect as much as possible in the present study, a 2-h face-to-face orientation session prior to randomization included a careful explanation that the effect of providing non-alcoholic beverages on alcohol consumption was unknown. We also asked participants in both groups to go about their daily lives as usual, with the exception of keeping a drinking diary. Finally, alcohol consumption and the consumption of non-alcoholic beverages were reported using a messaging app online. Given these measures, we consider it unlikely that the Hawthorne affected the study results. As such, provision of non-alcoholic beverages may be a new approach to behavioral modification that facilitates reduced alcohol consumption.

Although there was no significant difference in the intervention group between alcohol consumption at Week 12 and that at Week 20, the mean value did increase slightly, suggesting that the alcohol consumption-reducing effect of providing non-alcoholic beverages did not persist long term after the intervention was complete. However, the reduction of alcohol consumption over at least 20 weeks is considered beneficial in terms of public health.

The control group in this study also exhibited reduced alcohol consumption at Week 12, on average 2.7 g/day. This change may have been caused by the requirement to keep a drinking diary. According to a systematic review, self-monitoring of substance use disorder was effective in 29% of the analyzed studies, ineffective in 63%, and harmful in 8% [29]. Because people with alcoholism were excluded from the present study, the drinking diary may have effectively reduced alcohol consumption. Assuming that the effect in the control group was the result of keeping a drinking diary, the decreased alcohol consumption observed in the intervention group is speculated to result from the combination of providing non-alcoholic beverages and keeping a drinking diary. For this reason, it may be appropriate to estimate the alcohol consumption-reducing effect of providing non-alcoholic beverages to be 8.8 g/day (i.e., the observed 11.5-g reduction in this group minus the 2.7-g reduction in the control group). The cost of the 12-week intervention required to reduce alcohol consumption by 8.8 g/day per person was 19,658.5 yen (approximately 144.8 US dollars; 1,061,564 yen divided by 54 [participants]). Because it has been suggested that there is a negative association between the price of non-alcoholic beverages and the number of consumers of such beverages [30], a price increase is likely to result in fewer purchasers. Therefore, the results of this study, specifically the low dropout rate, may be attributable to the fact that non-alcoholic beverages were provided free of charge. Further research is necessary to determine how the results and dropout rate are affected when participants are required to pay the costs themselves.

Since the purpose of this study was to investigate whether increased availability of non-alcoholic beverages would change the amount of alcohol consumption, we first examined the effect of providing non-alcoholic beverages purchased by the research group. The fact that alcohol consumption decreased when these non-alcoholic beverages were provided to the participants indicates that this strategy may be useful as a public health policy (e.g., providing price incentives for non-alcoholic beverages) in the real world [3].

From a clinical point of view, it will be beneficial to examine whether alcohol use changes if individuals purchase non-alcoholic beverages themselves. However, as the non-alcoholic beverages delivered to the intervention group were selected from a list specified by the participants based on their own preferences, we believe that this study serves as a valuable foundation for future studies.

In this study, the number of participants differed between the two groups because of simple randomization, but there was no significant bias in basic attributes or baseline data, and the low percentage of dropouts (1.6%) contributed to the validity of the study. However, there are roughly five major limitations to this study. The first is that almost all participants were Japanese, and therefore, caution should be exercised when generalizing the results across different races. Information was collected on alcohol metabolism-related genes as well as on body size (height and body weight), and these data are considered to be helpful for interpreting the results of this study. The second limitation is the narrowly defined target population in this study. Since none of the participants had a history of alcoholism, it remains unknown whether the results of this study can be applied to individuals with alcoholism or those at high risk of other alcohol-related problems [20]. Therefore, future studies should examine the effect of providing non-alcoholic beverages to these populations, as well as the cost effectiveness of such programs. The third limitation is that the data on alcohol consumption and non-alcoholic beverage consumption were reported by participants themselves. While several objective blood biomarkers reflecting excessive drinking exists, there are some problems, such as large individual variation of the biomarkers and short half-life of them [31, 32]. Moreover, the longitudinal study requires many times of blood collection, which can increase drop-out our study. From these observations, we used only diary to obtain the amount of drinking alcoholic and non-alcoholic beverages. However, only self-reported data were analyzed and interpreting the results requires caution. The fourth limitation is that this study was conducted in an open-label manner. In this study, all participants were notified of their group assignment 2 weeks before the start of the intervention, due to the preparation time needed before providing them with the non-alcohol beverages of their choice. Although none of the baseline data differed significantly between the two groups, revealing group assignments may have affected baseline values and the main outcome of change in alcohol consumption. The last limitation is that this study may have included unmeasurable confounding factors. Thus, future studies should enroll larger populations.

## Conclusions

Our results suggested that providing free non-alcoholic beverages was associated with significantly reduced alcohol consumption. This effect persisted for 8 weeks after the completion of the intervention. Providing non-alcoholic beverages may be a strategic option for reducing alcohol consumption among people with excessive drinking.

### Supplementary Information

**Additional file 1:** **Figure S1.** Correlation between changes in alcohol consumption and non-alcoholic beverage consumption at Weeks 4, 8, 16, and 20 in the control and intervention groups. Panels A, C, E, and G indicate the relationships at Weeks 4, 8, 16, and 20, respectively, in the control group (all panels, n = 67). Panels B, D, F, and H indicate the relationships at Weeks 4, 8, 16, and 20, respectively, in the intervention group (all panels, n = 54).

**Additional file 2:** **Table S1.** Characteristics in the participants other than Table 1. **Table S2.** The number of populations of genetic polymorphisms in ADH1B and ALDH2 in the participants.

## Data Availability

Not applicable.

## Abbreviations

ADH1B: Alcohol dehydrogenase 1B
ALDH2: Aldehyde dehydrogenase 2
ANOVA: Analysis of variance
AQoLS: Alcohol Quality of Life Scale
AUDIT: Alcohol Use Disorders Identification Test
COVID-19: Coronavirus disease 2019
HED: Heavy episodic drinking
WHO: World Health Organization
