# Supplementary material for: Effect of provision of non-alcoholic beverages on alcohol consumption: a randomized controlled study
Source: BMC Med. 2023 Oct 2;21:379. doi: 10.1186/s12916-023-03085-1 (PMC10544561; doi:10.1186/s12916-023-03085-1)
Supplement: Supplementary file 2 — Additional file 2: Table S1. Characteristics in the participants other than Table 1. Table S2. The number of populations of genetic polymorphisms in ADH1B and ALDH2 in the participants. [file 12916_2023_3085_MOESM2_ESM.docx]

Supplemental Table 1 Participant characteristics not shown in Table 2.

|  |  | N = 123 |  | Intervention group  n = 54  (43.9%) |  | Control group  n = 69  (56.1%) |  | p |
| --- | --- | --- | --- | --- | --- | --- | --- | --- |
| Japanese (number of participants, %) |  | 122 (99.2) |  | 53 (98.2) |  | 69 (100.0) |  | 0.44^a^ |
| Height (cm, SD) |  | 165.6 (8.6) |  | 165.5 (8.3) |  | 165.7 (9.0) |  | 0.92^b^ |
| Body weight (kg, median, IQR) |  | 61.8 (22.2) |  | 65.4 (22.2) |  | 61.6 (19.9) |  | 0.82^c^ |
| Married |  | 89 (72.4) |  | 37 (68.5) |  | 52 (75.4) |  | 0.40^d^ |
| Employed (number of participants, %) |  | 119 (96.8) |  | 51 (94.4) |  | 68 (98.6) |  | 0.32^a^ |
| Household income |  |  |  |  |  |  |  | 0.30^a^ |
| ≤ 1 million yen |  | 1 (0.8) |  | 1 (1.9) |  | 0 (0.0) |  |  |
| 1 to 2 million yen |  | 0 (0.0) |  | 0 (0.0) |  | 0 (0.0) |  |  |
| 2 to 3 million yen |  | 4 (3.3) |  | 3 (5.6) |  | 1 (1.5) |  |  |
| 3 to 4 million yen |  | 9 (7.3) |  | 4 (7.4) |  | 5 (7.3) |  |  |
| 4 to 5 million yen |  | 16 (13.0) |  | 8 (14.8) |  | 8 (11.6) |  |  |
| 5 to 8 million yen |  | 29 (23.6) |  | 9 (16.7) |  | 20 (29.0) |  |  |
| 8 to 10 million yen |  | 26 (21.1) |  | 9 (16.7) |  | 17 (24.6) |  |  |
| ≥ 10 million yen |  | 38 (30.9) |  | 20 (37.0) |  | 18 (26.1) |  |  |
| Smoking history |  |  |  |  |  |  |  | 0.82^d^ |
| Smoker |  | 22 |  | 11 |  | 11 |  |  |
| Ex-smoker |  | 38 |  | 16 |  | 22 |  |  |
| Non-smoker |  | 63 |  | 27 |  | 36 |  |  |
| AQoLS (points, median, IQR) |  | 2.0 (4.0) |  | 3.0 (5.0) |  | 2.0 (3.0) |  | 0.092^c^ |
| Subjective view of health (number of participants, %) |  |  |  |  |  |  |  | 0.56^a^ |
| Very healthy |  | 33 (26.8) |  | 17 (31.5) |  | 16 (23.2) |  |  |
| Fairly healthy |  | 86 (69.9) |  | 35 (64.8) |  | 51 (73.9) |  |  |
| Not so healthy |  | 4 (3.3) |  | 2 (3.7) |  | 2 (2.9) |  |  |
| Not healthy |  | 0 (0.0) |  | 0 (0.0) |  | 0 (0.0) |  |  |

SD, standard deviation; IQR, interquartile range.

^a^ Fisher’s exact probability test, ^b^ t-test, ^c^ Mann–Whitney U test, ^d^ Chi-square test.

Supplemental Table 2 Numbers of genetic polymorphisms in *ADH1B* and *ALDH2* in study participants.

| Gene involved in alcohol metabolism |  | N = 122 |  | Intervention group  n = 54  (44.3%) |  | Control group  n = 68  (55.7%) |  | p |
| --- | --- | --- | --- | --- | --- | --- | --- | --- |
| *ADH1B* |  |  |  |  |  |  |  | 0.51^c^ |
| *1/*1 |  | 11 (9.0) |  | 3 (5.6) |  | 8 (11.8) |  |  |
| *1/*2 |  | 31 (25.4) |  | 15 (27.8) |  | 16 (23.5) |  |  |
| *2/*2 |  | 80 (65.6) |  | 36 (66.7) |  | 44 (64.7) |  |  |
| *ALDH2* |  |  |  |  |  |  |  | 0.075^c^ |
| *1/*1 |  | 98 (80.3) |  | 47 (87.0) |  | 51 (75.0) |  |  |
| *1/*2 |  | 24 (19.7) |  | 7 (13.0) |  | 17 (25.0) |  |  |
| *2/*2 |  | 0 (0.0) |  | 0 (0.0) |  | 0 (0.0) |  |  |

*ADH1B*, alcohol dehydrogenase 1B; *ALDH2*, aldehyde dehydrogenase 2.

^a^ Fisher 's exact probability test
